# Correction: PrP^ST^, a Soluble, Protease Resistant and Truncated PrP Form Features in the Pathogenesis of a Genetic Prion Disease

**DOI:** 10.1371/journal.pone.0133911

**Published:** 2015-07-20

**Authors:** Yael Friedman-Levi, Michal Mizrahi, Kati Frid, Orli Binyamin, Ruth Gabizon

Concerns have been raised about two of the figures published in this article (see: https://pubpeer.com/publications/23922744). The first concern was about Figure 2c ('middle right panel: The right 2 lanes look very similar') whilst the second was about Figure 3b ('PK-panel: A rectangle with a much lighter background than the rest of the blot appears to be visible in the first 4 lanes.').

PLOS Staff Editors contacted the authors for a response to these concerns and to request the original gels for these figures. The authors acknowledged that the figures contained some imperfections, which they suggested related to the scanning backgrounds.

The authors have declared that the figure imperfections have no effect on the results and conclusions of the study. They have provided the journal office with the original gel for Figure 3b and the original gel from a similar experiment for Figure 2c. The original gels and the published figures have been reviewed by a Section Editor (Prof. Per Westermark), who is satisfied with the authors' response to the concerns.

Please find the original gels included below as supporting information files.

## Supporting Information

S1 Gel Original Gel for Fig 3b. (TIF)

S2 Gel Original Gel for a similar experiment for Fig 2c. (TIF)
