# Supplementary figures and images for: Correction: PrPST, a Soluble, Protease Resistant and Truncated PrP Form Features in the Pathogenesis of a Genetic Prion Disease
Source: PLoS One. 2015 Jul 20;10(7):e0133911. doi: 10.1371/journal.pone.0133911 (PMC4507992; doi:10.1371/journal.pone.0133911)

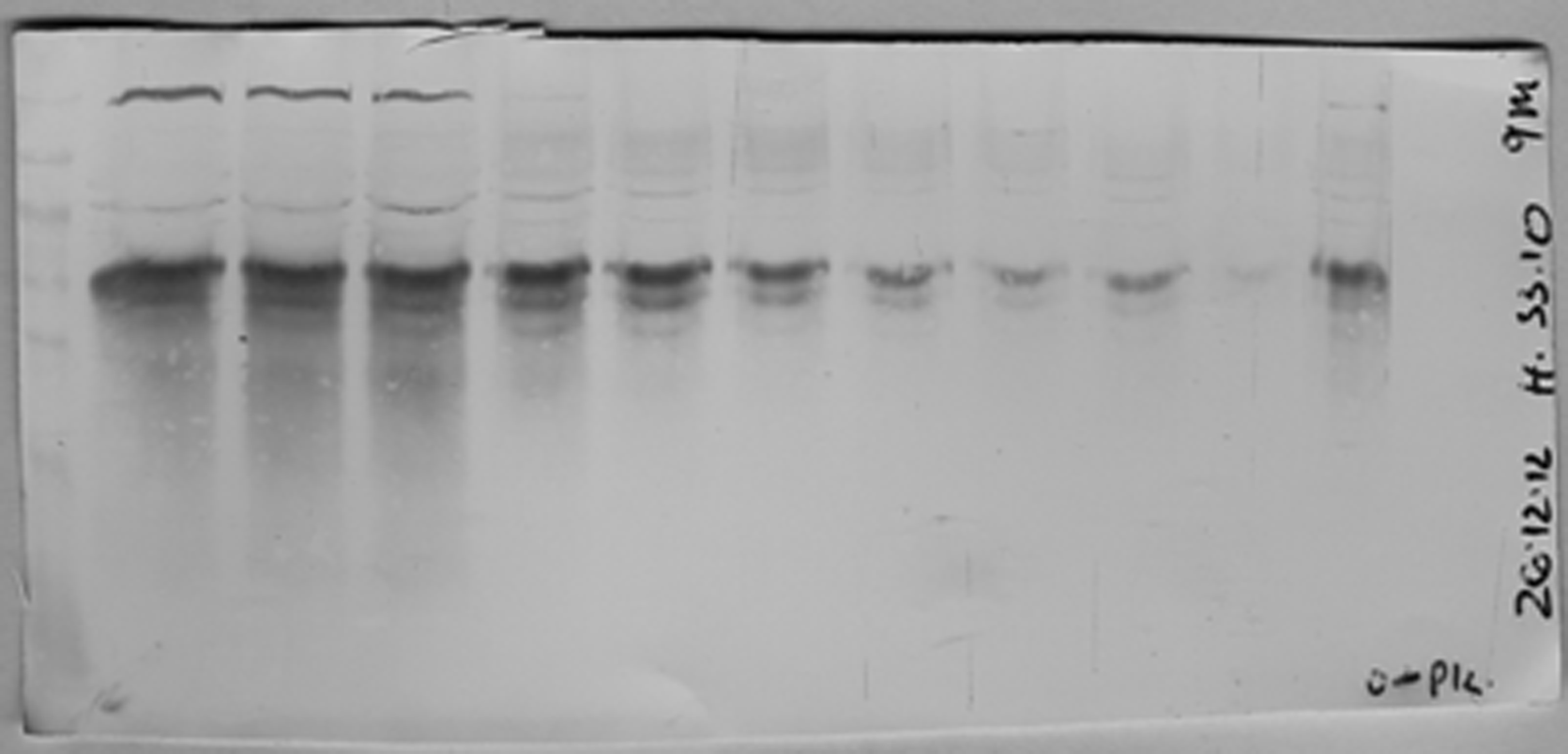

Supplement: S1 Gel — (TIF) [file pone.0133911.s001.tif]

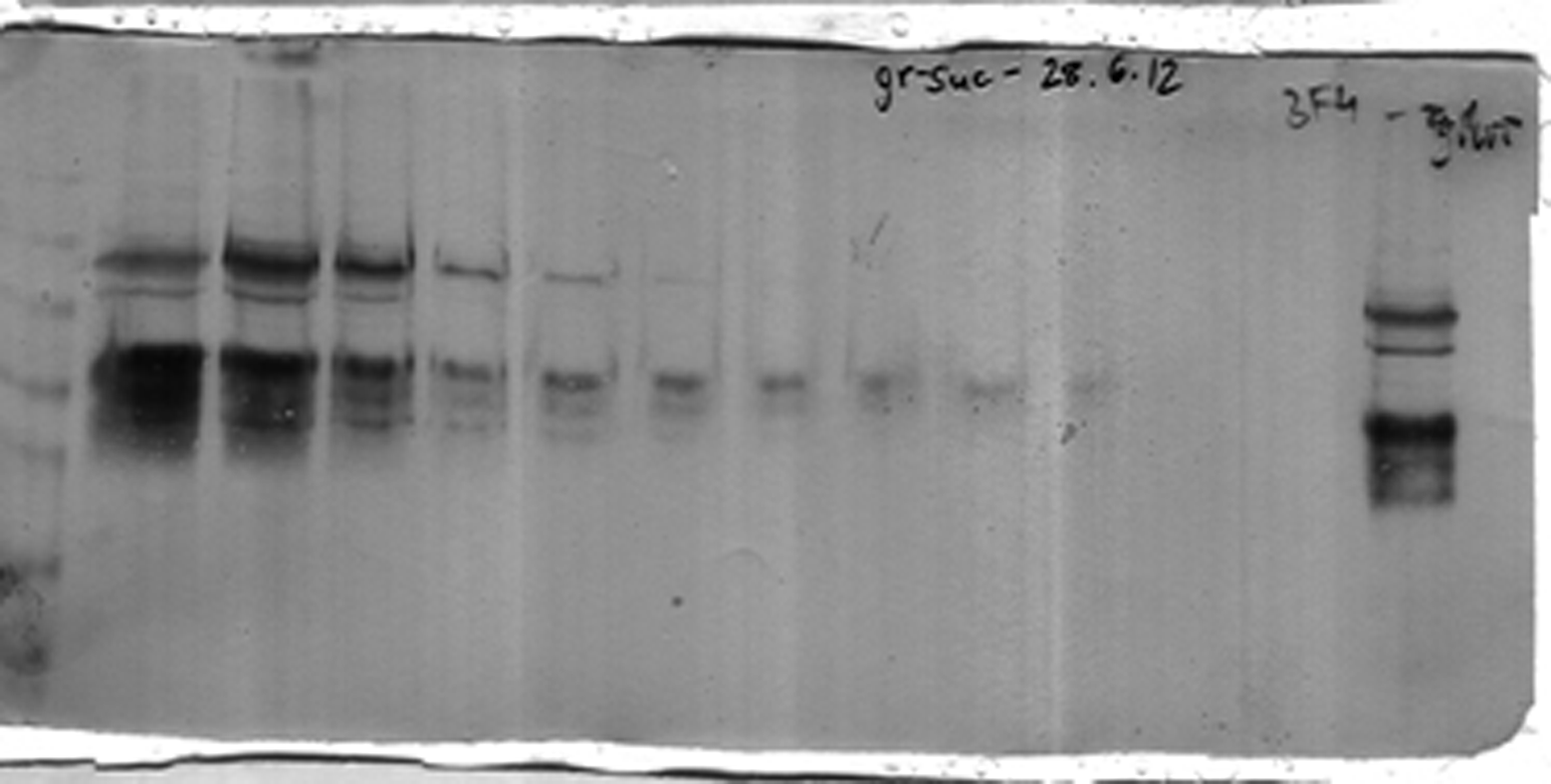

Supplement: S2 Gel — (TIF) [file pone.0133911.s002.tif]
